# Heart rate variability in hyperthyroidism on sub Saharan African patients: a case–control study

**DOI:** 10.1186/s13104-018-3922-4

**Published:** 2018-11-15

**Authors:** Eliane Ngassam, Marcel Azabji-Kenfack, Aurel T. Tankeu, Liliane Mfeukeu-Kuate, Chris-Nadège Nganou-Gnindjio, Camille Mba, Jean Claude Katte, Mesmin Y. Dehayem, Jean Claude Mbanya, Eugène Sobngwi

**Affiliations:** 1 National Obesity Center, Yaoundé Central Hospital, Yaoundé, Cameroon; 2 Faculty of Medicine and Biomedical Sciences, University of Yaoundé 1, Yaoundé, Cameroon

**Keywords:** Hyperthyroidism, Heart rate variability, Short-term, Africa

## Abstract

**Objective:**

We aimed to determine heart rate variability in freshly diagnosed untreated hyperthyroidism patients. We enrolled 10 patients (9 females) and 10 matched controls for sex and age. Each eligible patient underwent five different tests according to Ewing battery tests for cardiac autonomic dysfunction assessment. HRV was assessed during each maneuver and on 24 h using a continuous electrocardiogram with automatic estimation of SDNN, RMSSD, LF HF and HF/LH ratio. Results of tests were compared between hyperthyroidism patients and matched controls using the non-parametric test of Mann–Whitney.

**Results:**

Heart rate was significantly higher in patients with thyrotoxicosis (82.91 ± 10.99 vs 67.04 ± 6.80; 0.006) compared to their controls. On time-domain analysis, there was a trend towards reduction in SDNN (39.52 vs. 63.75; p = 0.2) as well as the RMSSD (30.44 vs 64.03; p = 0.09) in patients with hyperthyroidism. The frequency-domain analysis showed non-significant higher values for the LF (43.87 vs 38.85 ± 12.85; p = 0.8) and lower for the HF (32.54 vs 43.39; p = 0.3). Test’s results were mostly impaired in hyperthyroid patients and all patients presented abnormal results for parasympathetic activity. Untreated and recently diagnosed hyperthyroidism is associated to an altered parasympathetic activity in sub Saharan African patients.

## Introduction

Heart is a major target organ for thyroid hormone action and hyperthyroidism is characterized by a variety of clinical features that closely resemble those of catecholamine excess [1, 2]. These include palpitations, increase in resting heart rate due to sinus tachycardia but studies on catecholamine’s levels in hyperthyroidism revealed normal rate of plasma norepinephrine in thyrotoxicosis and additional data suggested that increased sensitivity to catecholamines cannot explain clinical manifestations of thyrotoxicosis in humans [3, 4]. Moreover, the increased of intrinsic heart rate in hyperthyroidism has been associated with an increased sympathetic autonomic activity and reduced parasympathetic tone due to a direct effect of hormone excess [5, 6]. Hyperthyroidism is therefore a sympatho-vagal imbalanced state, characterized by altered autonomic modulation of the heart rate [7]. Heart rate variability (HRV) reflects beat-to-beat changes in RR intervals, which are related to the ongoing interplay between two arms of the autonomic nervous system [8]. It is a noninvasive physiological marker used to assess autonomic nervous function [9]. Study of Heart rate variability (HRV) is an indicator of the balance between parasympathetic and sympathetic activity of the autonomic nervous system [10]. It is of great importance since reduced HRV signals a dysfunction in vagal activity and predicts an increased risk of sudden cardiac death and total cardiac mortality in some groups of patients such as those with myocardial infarction [10]. But studies of HRV in clinical hyperthyroidism are still scare in sub-Saharan African region. Our study aimed to determine short term HRV in freshly diagnosed and untreated hyperthyroidism in sub Saharan African patients.

## Main text

### Methods

#### Setting and population

We conducted a case–control study at the National Obesity Center of the Yaoundé Central Hospital on freshly diagnosed and untreated hyperthyroidism patients and their controls matched for sex and age. Patients who were already treated for hyperthyroidism or presenting a complication of this condition or preexisting cardiovascular disease were excluded.

#### Procedure and investigations

The study required two visits. At the inclusion visit, a detailed clinical exam and venous sample for biological investigations were performed to confirm the diagnosis of hyperthyroidism. For patients presenting clinical hyperthyroidism, blood samples were drawn for thyroid hormones measurement. These were free Thyroxin (FT_4_), free Triiodothyronine (FT_3_) and Thyroid Stimulating Hormon (TSH) allowing biological diagnosis. Following diagnosis confirmation, a resting electrocardiogram was done and patients presenting overt cardiac abnormality were excluded. The exploration visit was conducted in the morning in a quiet room. Blood pressure and heart rate were measured in the sitting position after a rest of at least 5 min using an automatic sphygmomanometer (BP-103iII; OMRON Colin Co., Tokyo, Japan) placed on the left arm raised itself at the heart level. Weight and height were respectively valued to the nearest 0.5 unit using a mechanical scale and a height gauge, then, body mass index (BMI) was calculated as weight divided by height squared.

#### Heart Rate Variability measurement

The measurement of HRV was done using a continuous ECG device (heart rate meter, Polar RS 800). Previously moistened electrodes were placed on the chest of patient at the level of the 5th rib in order to capture heartbeats. The corresponding watch recording data was placed on the patient’s left wrist. After installation of this equipment, the patient was stretched for 5 min, time for adaptation to this new material, before the beginning of different maneuvers. The device was then started.

To evaluate cardiac autonomic function, each patient went through 5 tests according to the Ewing’s standards battery testing to evaluate autonomic cardiac sympathetic and parasympathetic dysfunction as previously described by Ewing [11]. There were distributed as follows: heart rate response to Valsalva maneuver, heart rate response to deep breathing, blood pressure response to sustained handgrip, immediate heart rate response to standing and blood pressure response to standing. The three first tests helped us estimating parasympathetic abnormalities while the two remaining were used to appreciate sympathetic function.

The first test was done with the patient blowing in a manual pressure device until the needle reaches 40 mmHg; at this level, the patient should keep the needle for as long as he was able before releasing. This maneuver was performed twice at 5 min intervals. The time during which the patient had been able to maintain the needle to 40 mmHg was noted. Normally, during this maneuver, the patient exhibit a tachycardia characterized by a short space RR (RR minimum). After stopping, there is a bradycardia characterized by a long RR space (maximum RR) and RR maximum/minimum RR ratio (Valsalva ratio) is calculated.

The second test consisted of an ample breathing every 10 s for 1 min, for a total of 6 breaths and was performed 3 min after the end of the first one. During each cycle, the maximum and minimum HR was noted. Average maximal HR—average minimal HR, gave us the difference of HR during the maneuver. The maximum and minimum R–R intervals during each breathing cycle are measured and converted to beat per minute. The result is then expressed as the mean of the difference between maximum and minimum heart rates for the six measured cycles in beats a minute. The average difference of 3 successive cycles was considered.

The third test was the evaluation of blood pressure response to sustained handgrip. The patient had to squeeze a tennis ball for 2 min in the right hand. The diastolic BP was measured at rest and at the end of the maneuver. Normally, there is a sharp rise in blood pressure due to a heart-rate-dependent increase in cardiac output with unchanged peripheral vascular resistance.

But this increase is reduced in case of sympathetic abnormalities.

The fourth test was the estimation of immediate heart rate response to standing. During the change from lying to standing a characteristic immediate rapid increase in heart rate occurs and is maximal at about the 15th beat after standing. A relative overshoot bradycardia then occurs, maximal at about the 30th beat. The shortest R-R interval at or around the 15th beat and the longest R-R interval at around the 30th beat after standing was measured and characterized the heart rate response expressed by the 30:15 ratio.

Finally, the fifth test was evaluation of blood pressure response to standing. This maneuver consisted of a rapid change in the supine position to standing and this in less than 3 s.

After ending the last test, the patient returned home with functioning device which recorded HRV for the following 24 h.

Autonomic dysfunction was interpreted as follows: a total score ≤ 3 was considered normal autonomic function. A total score > 3 and < 8 was considered limited and a score ≥ 8 was considered abnormal. The results were then categorized into four groups: normal; early parasympathetic damage with results of one of the three tests of parasympathetic function abnormal; definite parasympathetic damage with results of at least two of the tests of parasympathetic function abnormal; and combined parasympathetic and sympathetic damage, where in addition to abnormal parasympathetic test results findings in one or both of the sympathetic tests are abnormal [11].

#### Statistical analysis

The calculations were performed using SPSS 12.0. Continuous variables are expressed as mean ± standard deviation. The Mann–Whitney test for independent samples was use with a significance set of p < 0.05 was considered as statistically significant.

### Results

#### General characteristics

Ten patients with newly diagnosed thyrotoxicosis and naïve to any treatment were enrolled in the study (9 females) and compared to ten controls matched for sex and age. The mean age of patients was 40.3 ± 10.9 years vs 40.9 ± 11.9 years for controls. The average BP of the patients was 126/68 mmHg vs 103/56 mmHg for controls (p = 0.006). The mean heart rate of patients was also higher than controls one (98 vs 68; p < 0.0001). In contrast, weight (61.8 vs 70.8 kg) and BMI (23.1 vs 27.05 kg/m^2^) of patients was lower than control but the difference remains non-significant (p = 0.2 and 0.1).

#### Clinical and biological signs of hyperthyroidism

Concerning clinical exam, fatigability and irritability were the two main complaints seen in 09/10 patients. These were followed by weight loss, asthenia, and palpitations (08/10 patients) while transit disorders (diarrhea/constipation) and bone pain represented the less frequent complaints. On physical exam, ends tremor was found in 9/10; sweating in 7/10 and exophthalmia in 6/10 patients.

#### HRV in both groups using Ewing battery tests

HRV measures showed an RR interval (0.74 ± 0.10 vs 0.91 ± 0.11; 0.006) and heart rate (82.91 ± 10.99 vs 67.04 ± 6.80; 0.006) significantly higher in patients with thyrotoxicosis. Ton time-domain analysis, SDNN (39.52 ± 19.54 vs. 63.75 ± 35.83; 0.205) as well as the RMSSD (30.44 ± 24.79 vs 64.03 ± 56.82; 0.097) were reduced in patients with hyperthyroidism but not significantly. The frequency-domain analysis showed higher values for the LF (43.87 ± 29.55 vs 38.85 ± 12.85; 0.845) and lower for the HF (32.54 ± 20.67 vs 43.39 ± 17.34; 0.38) respectively representing the sympathetic and parasympathetic alteration, however, they remained non-significant. The LF/HF ratio representing sympathovagal balance was not significantly increased in hyperthyroidism patient. Values of Ewing’s tests were mostly lowered in hyperthyroid patients but mostly in a non significant manner. Detailed results concerning responses to various maneuvers in patients presenting thyrotoxicosis are presented in Table 1 while Table 2 shows us comparison of these parameters between hyperthyroidism patients and matched controls.

**Table 1:** Comparison of HRV in hyperthyroidism patients and their matched controls during 2 h at rest

Variables	Hyperthyroidism patients	Controls	p (Mann–Whitney)
Mean RR (s)	0.8 ± 0.1	0.9 ± 0.11	0.006
Mean HR (1/min)	82.9 ± 11	67 ± 6.80	0.006
RMSSD (ms)	30.4 ± 24.8	64 ± 56.82	0.09
SDNN index	39.5 ± 19.5	63.7 ± 35.83	0.20
LF (n.u.)	43.9 ± 29.5	38.8 ± 12.85	0.84
HF (n.u.)	32.5 ± 20.6	43.3 ± 17.34	0.38
LF/HF	3.7 ± 5.35	1.05 ± 0.54	0.46

*RR* R to R interval, *HR* heart rate, *RMSSD* root-mean-square of successive differences, *SDNN* standard deviation of all normal R to R interval, *LF* low frequency, *HF* high frequency

**Table 2:** Comparison of HRV during Ewing battery tests in patients and their controls

Ewing test	Hyperthyroidism patients	Controls	p (Mann–Whitney)
HR response to Valsalva maneuver	1.27 ± 0.15	1.31 ± 0.19	0.65
Heart rate response to deep breathing	11.71 ± 6	11.34 ± 4.74	0.93
Blood pressure response to sustained handgrip	1.17 ± 0.11	1.20 ± 0.1	0.59
Immediate heart rate response to standing	2.67 ± 4.50	3.20 ± 3.01	0.58
Blood pressure to standing	− 5.00 ± 7.13	− 7.40 ± 8.90	0.50

### Discussion

This study aimed to determine HRV in a group of sub Saharan African patients with recently diagnosed hyperthyroidism and untreated. We found that heart rate was significantly increased in thyrotoxicosis patients during resting time. All patients having thyrotoxicosis presented signs of parasympathetic dysfunction on at least one of the Ewing battery test. Heart response to deep breathing was the most discriminative one. Overall parasympathetic activity was altered in hyperthyroidism group.

Regarding the interpretation of the results of different maneuvers, we found that on the Valsalva maneuver, all patients presenting hyperthyroidism (100%) drooled borderline and/or abnormal values while 20% of controls had completely normal tests. The heart response to deep breathing turned out to be the most discriminative classifying all patients with thyrotoxicosis as abnormal against only 10% in controls. Although absolute measures of HRV were not significantly lowered in hyperthyroidism, proportion of patients with thyrotoxicosis presenting an alteration on HRV during each maneuver was much higher than that of controls. All patients with hyperthyroidism had abnormal results on at least one of the five tests and this was the heart rate response to deep breathing which is used to assess parasympathetic cardiac autonomic dysfunction. These results are similar to others studies which found an alteration of HRV in hyperthyroidism patients, more marked on parasympathetic component except the fact that most of these studies found a significantly decrease of HRV measured by RMSSD and SDNN [10, 12, 13]. In contrast, the difference found on these parameters in our study was non-significant. This could be attribute to a small sample size but some studies using the same sample size found a significantly lower SDNN and RMSSD [14]. Moreover, even subclinical hyperthyroidism has been found associated to a significant decreased in HRV [12]. Since few have been made on African population concerning HRV and hyperthyroidism and taking into account the fact that the African population usually presents different features of cardiovascular diseases than others populations, we believe that these findings could suggest a less decrease of HRV in our sub Saharan population but there is a need of more studies with greater sample size and using others methods of HRV measurement to draw such conclusions.

Freshly diagnosed sub Saharan African patients presenting hyperthyroidism naïve to all treatment have an altered heart response to deep breathing suggesting a slight alteration in HRV more marked on parasympathetic.

### Limitation

The main limitation of this study is the small sample size limiting inferences. However, considering the low prevalence of hyperthyroidism in our context and the few existing studies on the subject, our results provide new and important information and should serve as a basis for the design of larger studies.

## Abbreviations

BP: blood pressure
FT3: free triiodothyronine
FT4: free thyroxin
HR: heart rate
HRV: heart rate variability
HF: high frequency
LF: low frequency
ms: milliseconds
RMSSD: root-mean-square of successive differences
SDNN: standard deviation of all normal R–R intervals
TRH: thyroid releasing hormone
TSH: thyroid stimulating hormone

## References

[1] Kahaly GJ, Dillmann WH (2005). Thyroid hormone action in the heart. Endocr Rev.

[2] Burggraaf J, Tulen JH, Lalezari S, Schoemaker RC, De Meyer PH, Meinders AE (2001). Sympathovagal imbalance in hyperthyroidism. Am J Physiol Endocrinol Metab.

[3] Liggett SB, Shah SD, Cryer PE (1989). Increased fat and skeletal muscle beta-adrenergic receptors but unaltered metabolic and hemodynamic sensitivity to epinephrine in vivo in experimental human thyrotoxicosis. J Clin Invest..

[4] Coulombe P, Dussault JH, Walker P (1977). Catecholamine metabolism in thyroid disease. II. Norepinephrine secretion rate in hyperthyroidism and hypothyroidism. J Clin Endocrinol Metab.

[5] Valcavi R, Menozzi C, Roti E, Zini M, Lolli G, Roti S (1992). Sinus node function in hyperthyroid patients. J Clin Endocrinol Metab.

[6] Petretta M, Bonaduce D, Spinelli L, Vicario ML, Nuzzo V, Marciano F (2001). Cardiovascular haemodynamics and cardiac autonomic control in patients with subclinical and overt hyperthyroidism. Eur J Endocrinol Eur Fed Endocr Soc..

[7] Chen J-L, Chiu H-W, Tseng Y-J, Chu W-C (2006). Hyperthyroidism is characterized by both increased sympathetic and decreased vagal modulation of heart rate: evidence from spectral analysis of heart rate variability. Clin Endocrinol (Oxf).

[8] Cygankiewicz I, Zareba W (2013). Heart rate variability. Handb Clin Neurol..

[9] Min KB, Min J-Y, Paek D, Cho S-I, Son M (2008). Is 5-minute heart rate variability a useful measure for monitoring the autonomic nervous system of workers?. Int Heart J..

[10] Falcone C, Matrone B, Bozzini S, Guasti L, Falcone R, Benzi A (2014). Time-domain heart rate variability in coronary artery disease patients affected by thyroid dysfunction. Int Heart J..

[11] Ewing DJ, Clarke BF (1982). Diagnosis and management of diabetic autonomic neuropathy. Br Med J Clin Res Ed..

[12] Kaminski G, Makowski K, Michałkiewicz D, Kowal J, Ruchala M, Szczepanek E (2012). The influence of subclinical hyperthyroidism on blood pressure, heart rate variability, and prevalence of arrhythmias. Thyroid Off J Am Thyroid Assoc..

[13] Shuvy M, Arbelle JE, Grosbard A, Katz A (2008). A simple test of one minute heart rate variability during deep breathing for evaluation of sympatovagal imbalance in hyperthyroidism. Isr Med Assoc J IMAJ..

[14] Barczyński M, Tabor S, Thor P (1997). Evaluation of autonomic nervous system function with heart rate variability analysis in patients with hyperthyroidism and during euthyroidism after pharmacologic and surgical treatment. Folia Med Cracov.

